# *Streptococcus pyogenes* strains associated with invasive and non-invasive infections present possible links with *emm* types and superantigens

**DOI:** 10.22038/IJBMS.2019.38635.9164

**Published:** 2020-01

**Authors:** Rao Muhammad Abid Khan, Sana Anwar, Zaid Ahmed Pirzada

**Affiliations:** 1 Department of Microbiology, University of Karachi, Karachi-75270, Pakistan; 2 Liaquat National Hospital, Karachi-75270, Pakistan

**Keywords:** GAS, Exotoxin, Invasive pathogens, Antibiotic resistance, MIC

## Abstract

**Objective(s):**

*Streptococcus pyogenes*, a notorious human pathogen is responsible to cause a wide range of infections varies from superficial common clinical illness to severe and life threatening infections. To our knowledge this is the first report exploring the *emm* types and superantigen/exotoxin gene profile of *S. pyogenes* from Pakistan.

**Materials and Methods:**

A total of 89 *S. pyogenes* strains were collected predominantly from throat swabs followed by pus, tissues and wound swabs. Profile of five superantigen genes speA, speB, speC, speF and ssa was screened for all the *emm* types.

**Results:**

Extensive heterogeneity among *S. pyogenes* strains was indicated, revealing 34 different *emm* types/ subtypes. The most prevalent *emm* types were *emm68 *and *emm104*. Some of the *emm* types were exclusively isolated from invasive infections while others were isolated only from non-invasive infections indicating the possible link between *emm* types and invasive/ noninvasive infections. Similarly, erythromycin-resistant strains mainly belonged to three particular *emm* types. Multiplex PCR analysis indicated the presence of speB 100%, speF 76%, speC 20%, speA 18% and ssa 15%. Interestingly, superantigen genes speC and speA were mainly associated with invasive infections. Among the five superantigens tested, one strain of *emm12* harbored all the analyzed exotoxin genes, while 4 strains carried 4 superantigen genes.

**Conclusion:**

*S. pyogenes* clones associated with invasive and non-invasive infections in Pakistan present differences in *emm* types, superantigens and antimicrobial resistance. The present data indicates the possible link between particular genetic lineage of a bacterium with the manifestation of the infection.

## Introduction

Group A Streptococcus (GAS) associated infections occur more commonly in developing countries compared to the developed countries. Six hundred and sixteen million cases of pharyngitis, 111 million cases of pyoderma and at least 517,000 deaths due to severe invasive diseases and sequelae were reported as the global burden due to GAS diseases since 2005 (1, 2). Invasive diseases are caused by the strains isolated from otherwise sterile body sites e.g. blood, cerebrospinal fluid, joints, pleural, peritoneal, or pericardial fluids or nonsterile body sites like wounds associated with streptococcal toxic shock syndrome (STSS) or necrotizing fasciitis (NF). Noninvasive GAS are isolated from patients suffering from pharyngitis, scarlet fever, erysipelas, and impetigo (3, 4). STSS was first reported in the late 1980s and have been the cause of high mortality rate ranging from 30% to 70% (5-7) and NF also called as flesh eating disease involves deeper skin layers and tissues (8, 9). Alarmingly, since late 1980s there has been a worldwide re-emergence of the severe forms of* Streptococcus pyogenes *infections, particularly NF and STSS (6, 10, 11).

*S. pyogenes* virulence factors can be divided into two groups: surface exposed virulence factors and secreted virulence factors. Among many factors involved in the pathogenesis of *S. pyogenes*, M protein and Streptococcal pyrogenic exotoxins (Spe) which exhibit the properties of superantigens are the most important ones (12). 

M protein is a major surface virulence (and adhesion) factor of *S. pyogenes* and is involved in various stages of GAS pathogenesis including adhesion, internalization, immune evasion and tissue invasion. It confers resistance to phagocytosis and killing by polymorphonuclear leukocytes by binding to complement control factors and other host proteins to prevent activation of the alternate complement pathway (13-15). M protein is encoded by *emm* gene which exhibits marked variability in its 5’ hypervariable region and forms the basis for* emm* genotyping (16). There are more than 100 different *emm* types reported to date (17). Epidemiology of virulent *S. pyogenes* strains from 10 countries revealed *emm*1, *emm*28, *emm*3, *emm*89, *emm*87, *emm*12, *emm*4, *emm*83, *emm*81 and *emm*5 as the most prevalent strains, particularly *emm*1 and *emm*3 were associated with STSS and NF (18). The distribution of *emm* gene considerably varies geographically and has been reported as an important surveillance tool for understanding the dynamics of GAS infections, its transmission, local epidemiology and indigenous vaccine development (19, 20). 

Among extracellular virulence factors superantigens (SAgs) are secreted proteins, which possibly contribute towards the pathogenesis of severe and invasive infections. SAgs/ exotoxins are the most potent T-cell mitogens reported so far (21). More than 40 bacterial SAgs have been reported in literature and a total of 12 antigenically distinct extracellular SAgs have been described in GAS to date which include streptococcal pyrogenic exotoxins (Spes) A, C, G-M*, *the streptococcal superantigen (Ssa) and streptococcal mitogenic exotoxin (SmeZ) 1 and 2 (22-25). Two proteins SpeB and SpeF (also known as mitogenic factor), previously described as SAgs, infact share the properties of cysteine protease and DNase respectively and therefore are not regarded as true SAgs (26, 27). SAgs are roughly 25 kDa secretory proteins that are implicated in the pathogenesis of GAS infections, including scarlet fever, STSS and rheumatic fever. SAgs have the unique ability to cross-link class II major histocompatibility complexes on antigen-presenting cells resulting in activation of up to 20% of T cells. This unspecific massive T-cell proliferation causes release of large amount of the cytokines. In addition to cytokine production and T-cell proliferation, SAgs are capable of inducing cytotoxicity towards target cells (28). These exotoxins also induce pyrogenicity, cause capillary leakage, activation of complement, coagulation and fibrinolytic cascades, leading to hypotension through cytokine release leading to shock, multi-organ failure and death. SAgs-coding genes among GAS are usually associated with bacteriophage vectors, except for *speG, speJ* and *smeZ *that are believed to be chromosomally encoded (22, 29). As SAgs SpeA, SpeC, SpeH, SpeI, SpeK, SpeL, SpeM, and SSA are encoded on prophage (30), therefore can be easily transduced among strains. Certain SAgs are associated with certain *emm* types. Interestingly, a relationship among SAgs and *emm* gene types with invasive infections has been reported (31-33).

*S. pyogenes* was thought to be sensitive to a large number of antibiotics. Although penicillins and macrolides have remained the drugs of choice for the treatment of streptococcal infections, but in the last decade GAS drug resistant strains have been reported from worldwide (including Europe and Asia) (33-36). In addition, clindamycin is considered efficient for invasive and severe streptococcal infections and fluoroquinolones can also be used, which shows the promising results during treatment (37). Many reports from developing and developed countries have shown an increase in resistance against macrolides and other antibiotics depending upon the geographical locations of the *S. pyogenes *strains, however only a few drug resistance reports against erythromycin have been published from Pakistan (38-41).

No data was available pertaining to the minimum inhibitory concentration of antibiotics, distribution of *emm* types and exotoxin profile of *S. pyogenes* isolates from Pakistan, therefore the present study was undertaken to achieve the above objectives and to fill the gap in scientific knowledge.

## Materials and Methods

***Collection of S. pyogenes strains***

 This study was undertaken at the Department of Microbiology, University of Karachi, Pakistan. Bacterial strains and patient’s basic information was obtained from different pathological laboratories, medical centres and tertiary care hospitals of Karachi. *S. pyogenes* strains were procured isolated from various clinical specimens like throat swabs, pus, blood, wounds, tissues, body fluids, urine and synovial fluid. 

***Purification and final identification of S. pyogenes strains***

All the procured *S. pyogenes* strains were purified on sheep blood agar (to get the pure culture) and were systematically identified by the routine parameters according to Bergey’s manual of determinative bacteriology diagnostic tests like catalase test, beta hemolysis, bacitracin sensitivity test etc. The confirmation of the strains was done by Lancefield grouping Kit (Oxoid, USA) while ABIS (online-advanced bacterial identification software) (www.tgw1916.net/bacteria_logare.html) was also referred for the final identification.

***Determination of minimum inhibitory concentration (MIC)***

Macrodilution technique was performed using tryptone soya broth to determine MIC (42). For the interpretation of MIC, CLSI guidelines (2015) were referred for breakpoints. All the experiments were carried out in triplicate to get the average values.

***emm typing of S. pyogenes strains***

*S. pyogenes *strains were analyzed by sequencing the 5’ end of *emm* gene, following the PCR protocols set by the Center for Disease Control and Prevention (CDC; http://www.cdc.gov/ncidod/biotech/strep/protocols.html) using the primer 1, 5-TAT TCG CTT AGA AAA TTA A-3 and primer 2, 5-GCA AGT TCT TCA GCT TGT TT-3. The *emm* gene sequences were identified by the homology analysis of sequences in the CDC database http://www.cdc.gov/ncidod/biotech/strep/strepblast.htm and http://www.ncbi.nlm.nih.gov/BLAST/Blast.cgi (30, 43). 

***Superantigen gene profile***

Profile of five superantigen genes *speA*, *speB*, *speC*, *speF* and *ss*a was screened for all the *emm* types by PCR analysis with the set of primers as already described (30). Amplification of all the genes was performed with an initial 3 min denaturation at 94 ^o^C, followed by 30 cycles of denaturation at 94 ^o^C for 60 sec, 60 sec of annealing temperatures standardized in the laboratory for multiplex PCR and 60 sec of extension at 72 ^o^C with a final extension step at 72 ^o^C for 7 min.

## Results

***Collection of S. pyogenes strains***

A total of 89 *S. pyogenes* out of 189 β-hemolytic streptococci procured (from different clinical specimens) were purified, identified and confirmed. Majority of the *S. pyogenes* were isolated from throat swabs 43 (48%), followed by pus 20 (22%), tissues, wound swab 9 (10%) each and blood 4 (4%). Recovery percentile from other specimens like body fluids 2 (2%), urine 1 (1%) and synovial fluid 1 (1%) was very low. 

***Determination of MIC***

The MIC analysis exhibited the highest level of resistance against clindamycin (1024 µg/ml) and azithromycin (512 µg/ml). Substantial level of resistance was observed for clarithromycin (128 µg/ml) and ciprofloxacin (8 ug/ml; Table 1), however all strains of *S. pyogenes* were found sensitive to penicillin and co-amoxiclav.

***emm typing of S. pyogenes strains***

**Figure 1:**
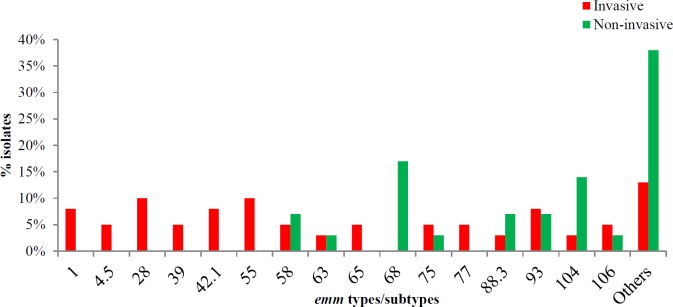
Distribution of *emm* types among 39 invasive and 29 non-invasive isolates

The *emm* gene sequencing was obtained for a total of 68 *S. pyogenes* strains. Among these strains 34 different *emm* types were identified indicative of a high degree of heterogeneity (Figure 1). The most prevalent* emm* types included *emm*68 and *emm*104 (11% each), followed by *emm*1, *emm*28, *emm*58 and *emm*75 (6% each), *emm*39, *emm*42*, emm*55*, emm*80*, emm*88 (4% each) and *emm*2, *emm*4.5*, emm*12*, emm*18*, emm*60*, emm*63*, emm*81*, emm*82.1*, emm*83*,* *emm*90*, emm*91*, emm*100*, emm*102 (2% each).

Overall, 22 different *emm* types were found among invasive strains; among which 15 *emm* types were exclusively found in invasive isolates. On the other hand, noninvasive isolates comprised 19 different *emm* types while 12 *emm* types were only found in noninvasive isolates. However, 7 *emm* types were present in both the invasive and the noninvasive specimens. Predominantly, *emm*55, *emm*28, *emm*1, *emm*42.1 were only traced in invasive infections while *emm*68 was only traced in noninvasive infections. Besides, *emm*58*, emm*75*, emm*93*, emm*88.3 and *emm*104 were isolated from both invasive and noninvasive infections; however, *emm*104 was more common among noninvasive compared to the invasive infections (Figure 1). 

The erythromycin-resistant strains comprised of 6 different *emm* types, with 70% of the isolates belonging to 3 predominant *emm* types i.e.* emm28* (30% of the isolates), *emm*75 and *emm*39 (20% each). The rest of the erythromycin-resistant isolates belonged to *emm*83, *emm*88 and *emm*4.5 types (10% each).

***Superantigen gene profile***

A total of 74 *S. pyogenes* strains were analyzed for the presence of *speA, speB, speC, speF* and *ssa* by multiplex PCR. The *speB* gene was present in all the strains whereas* speF* was present in 76% of the strains. Superantigen genes *speC, speA* and *ssa* were present in 20%, 18% and 15% of the *S. pyogenes* strains respectively. Interestingly, *speC* superantigen gene was predominantly (93%) observed in the invasive *S. pyogenes* strains, while *speA* was also found associated with 62% invasive infections (Table 2).

**Figure 2:**
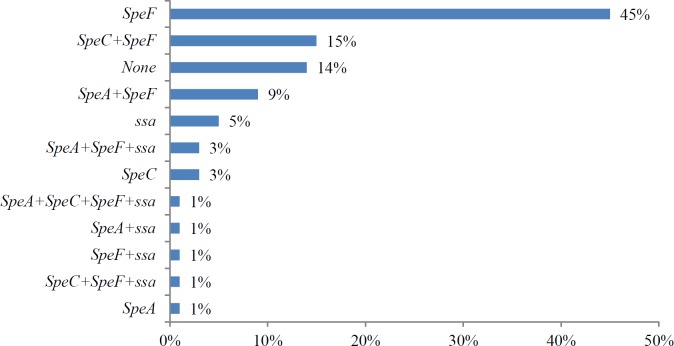
Distribution of 12 superantigen patterns

**Figure 3:**
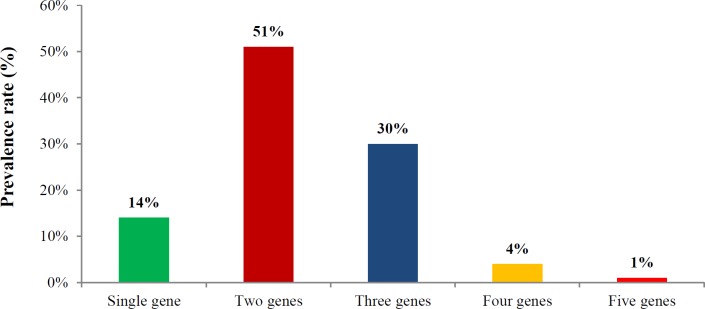
Frequency of exotoxins genes among *Streptococcus pyogenes*

Altogether, 12 different exotoxin patterns were observed in all the strains (Figure 2). The majority of the *S. pyogenes *strains exhibited 2 (51%) or three (30%) exotoxin genes whereas 4 strains (4%) carried 4 superantigen genes, while 14% strains carried one of the superantigen genes among them (Figure 3). Significantly, one strain belonging to *emm*12 possessed all the tested five exotoxin genes. Four *S. pyogenes *strains *emm*4.5,* emm*39, *emm*39.1 and *emm*65 carried four exotoxin genes and *emm*1*, emm*28*, emm*42.1*, emm*55*, emm*63*, emm*75*, emm*82.1 and *emm*100 harbored three exotoxin genes (Table 3). 

## Discussion

To our knowledge, the present investigation is the first comprehensive study that has shed light on the prevalence, chemotherapy and pathogenesis of *S. pyogenes* from Pakistan. In this study, although *S. pyogenes* were mainly isolated from throat swabs yet almost 50% of the *S. pyogenes* strains importantly have been isolated from invasive specimens like pus, wound, tissues, blood, body fluids hence *S. pyogenes* is not only responsible for the superficial infections but equally contributes to the invasive infections in Pakistan. 

Bacitracin susceptibility is used for the presumptive identification of GAS isolates, however it was observed that 16 (18%) strains of the *S. pyogenes* were resistant to bacitracin diagnostic disc. Similar phenomenon has also been reported from other countries (44, 45).

M protein is a major surface virulence factor. Sequencing of the hypervariable region of *emm* gene encoding M protein has been used as the gold standard for the epidemiological surveillance of the infections caused by *S. pyogenes*. This is the first document reporting the *emm* type prevalence from Pakistan indicating the great heterogeneity among *S. pyogenes* strains revealing 34 different* emm* types/subtypes. The most prevalent *emm* types included *emm*68 and* emm*104 (11% each) followed by *emm*1*,* *emm*28*, emm*58 and *emm*75 (6% each). A previous study from Denmark reported 29 different *emm* types with predominant *emm* types *emm*28 and* emm*1 (51%) (46). Another more recent study from USA reported 26 different *emm* types with predominant *emm* types *emm*48*, emm*89*,* *emm*4*, emm*12*, emm*75*, emm*1 etc (47-49). According to other studies the *emm*1*, emm*4*, emm*12 and *emm*28 were the predominant *emm* types from Austria (50), while *emm* 11 was reported to be the most common type from India (37, 51, 52).

In the current study, the *emm*1, *emm*28, *emm*42.1 and *emm*55 types were exclusively isolated from invasive infections while *emm*68 was only isolated from noninvasive infections thereby indicating a possible relationship between *emm* types and invasive/ noninvasive infections. It is known that *emm*1 is widely associated with streptococcal invasive diseases (49, 53, 54). A recent study from Japan reported the emergence of STSS associated 90% mainly with *emm*1, followed by *emm*89, *emm*12, *emm*28, *emm*3 and *emm*90 (55), while *emm*102 has also been associated with STSS in southern Taiwan (26). A number of similar *emm* types have been identified in the present study but it needs to design further comprehensive clinical, epidemiological and molecular research studies which can establish *emm* types association with STSS in Pakistan.

Superantigens secreted by *S. pyogenes* play an important role in the pathogenesis of the infections caused by this pathogen. Studies have suggested that certain superantigens are associated with invasive infections specially phage associated SAgs can easily be transferred to other noninvasive streptococcal strains. The profiling of SAgs genes was done in this study, whereby the prevalence of superantigen genes remained as *speF* (76%), s*peC* (20%), s*peA* (18%) and *ssa* (15%). In comparison a study from Norway reported superantigen prevalence s*peA* (75%), s*peC* (31%) and* ssa* (29%) (56), while exotoxin gene prevalence from India reported as *speF* (91%), s*peC* (24%), *ssa* 16.5% and s*peA* (7.5%) (51).

In the present study *speC* was significantly (93%) associated with invasive infections and *speA* was associated with 62% of the invasive infections. According to a previous study, *emm*77 was originated from invasive samples while *emm*1 and *emm*12 from noninvasive isolates (57). Although, the presence of a particular SAg gene cannot be exclusively related with the development of STSS, SAgs encoded by phage such as *speA* and *speC* have commonly been associated with invasive infections (58-60). 

During the present study one strain isolated from the throat swab belonging to *emm*12 harbored all the five analyzed exotoxin genes, thus it can be a potentially virulent strain. Recently, it has been reported that *emm*12 has been associated with the emergence of scarlet fever, toxin acquisition and multidrug resistance in Hong Kong and Australia (61-63). Significant association of *speA *with *emm1* and *speC* with *emm*12 has also been reported from Brazil (22)*.* Similarly, 4 other strains carried 4 SAgs genes, altogether indicating the presence of virulent strains which can potentially transmit these virulent genes to other strains as well as to other groups of non-pathogenic streptococci. On the other hand the majority of strains without having SAg genes belonged to the noninvasive strains group. 

The knowledge about the increased macrolide and lincosamide resistance among *S. pyogenes* reported by the current research study would benefit medical practioners to more accurately prescribe patients while dealing with streptococcal infections and monitoring the emergence of drug resistance. 

**Table 1:** Minimum inhibitory concentration (MIC)of antibiotics against *Streptococcus pyogenes*

**Antibiotic Group**	Antibiotics	MIC Range (µg/ml)	MIC_50_ (µg/ml)	MIC_90_ (µg/ml)	Breakpoint (Sensitive/ Resistance)	Level of resistance (No. of times)
**Lincosamide**	Clindamycin	4- >1024	64	>1024	<0.25/>1	1024
**Macrolides**	Azithromycin	4 - >1024	32	512	<0.5/>2	512
	Clarithromycin	1 - 128	2	32	<0.25/>1	128
**Quinolone**	Ciprofloxacin	4- 32	8	32	<1/>4	8
**MIC break points are according to CLSI, 2015 and NCCLS**						

(Other include *emm* types identified only in one sample i.e. *emm*2, *emm*3, *emm*12, *emm*18.2, *emm*48, *emm*60, *emm*80, *emm*81, *emm*82.1, *emm*83, *emm*90, *emm*91, *emm*100, *emm*102 and *emm*std

**Table 2:** Exotoxin genes distribution among invasive and noninvasive *Streptococcus pyogenes* infections

	speA n (%)	speB n (%)	speC n (%)	speF n (%)	ssa n (%)
*Invasive infections (41)*	8 (62)	41 (55)	14 (93)	30 (54)	6 (55)
*Non-invasive infections (33)* *Total infections (74) *	5 (38) 13 (18)	33 (45) 74 (100)	1 (7) 15 (20)	26 (46) 56 (76)	5 (45) 11 (15)

**Table 3:** Correlation of *emm* types/subtypes with exotoxin genes

*emm* types/subtypes	*speA*	*speB*	*speC*	*speF*	*ssa*
*emm1*	**+**	**+**		**+**	
*emm1.2*	**+**	**+**		**+**	
*emm2*		**+**	**+**		
*emm3*	**+**	**+**			**+**
*emm4.5*	**+**	**+**		**+**	**+**
*emm12*	**+**	**+**	**+**	**+**	**+**
*emm18.2*		**+**			
*emm28*		**+**	**+**	**+**	
*emm39*		**+**	**+**	**+**	**+**
*emm39.1*		**+**	**+**	**+**	**+**
*emm42.1*		**+**	**+**	**+**	
*emm48*		**+**			
*emm55*		**+**	**+**	**+**	
*emm58*		**+**		**+**	**+**
*emm60*		**+**		**+**	
*emm63*	**+**	**+**		**+**	
*emm65*	**+**	**+**		**+**	**+**
*emm68*		**+**		**+**	
*emm68.1*		**+**		**+**	
*emm75*		**+**	**+**	**+**	
*emm77*		**+**		**+**	
*emm80*		**+**		**+**	
*emm81*		**+**		**+**	
*emm82.1*		**+**	**+**	**+**	
*emm83*		**+**		**+**	
*emm88.3*		**+**		**+**	
*emm90*		**+**		**+**	
*emm91*		**+**		**+**	
*emm93*	**+**	**+**		**+**	
*emm100*		**+**	**+**	**+**	
*emm102*		**+**		**+**	
*emm104*		**+**		**+**	
*emm106*		**+**	**+**	**+**	
*std432*		**+**			**+**

## Conclusion

As the links do exist between particular genetic lineage and the type of infection, hence the genetic makeup of the bacteria play an important role in determining the outcome of the interaction between pathogen and its host. Our data has signified the multifactorial nature of *S. pyogenes* strains in a particular infectious manifestation. 

## References

[1] Efstratiou A, Lamagni T, Ferretti JJ, Stevens DL, Fischetti VA Epidemiology of Streptococcus pyogenes. Streptococcus pyogenes: Basic Biology to Clinical Manifestations.

[2] Sims Sanyahumbi A, Colquhoun S, Wyber R, Carapetis JR, Ferretti JJ,, Stevens DL,, Fischetti VA Global disease burden of group A Streptococcus. Streptococcus pyogenes : Basic Biology to Clinical Manifestations.

[3] Strus M, Heczko PB, Golinska E (2017). The virulence factors of group A Streptococcus strains isolated from invasive and noninvasive infections in Polish and German centre 2009-2011. Eur J Clin Microbiol Infect Dis.

[4] Matsumoto M, Yamada K, Suzuki M, Adachi H, Kobayashi S, Yamashita T (2016). Description of the pathogenic features of Streptococcus pyogenes isolates from invasive and noninvasive diseases in Aichi, Japan. Jpn J Infect Dis.

[5] Yoshizawa S, Matsumura T, Ikebe T, Ichibayashi R, Fukui Y, Satoh T (2019). Streptococcal toxic shock syndrome caused by beta-hemolytic streptococci: Clinical features and cytokine and chemokine analyses of 15 cases. J Infect Chemother.

[6] Hua CZ, Yu H, Yang LH, Xu HM, Lyu Q, Lu HP (2018). Streptococcal toxic shock syndrome caused by Streptococcus pyogenes: a retrospective study of 15 pediatric cases. Zhonghua Er Ke Za Zhi.

[7] Bartos H, Fabianova L, Dlouhy P (2018). Streptococcal toxic shock syndrome - a life-threatening condition caused by various Streptococcal species. Epidemiol Mikrobiol Imunol.

[8] Liu TJ, Tai HC, Chien KL, Cheng NC (2019). Predisposing factors of necrotizing fasciitis with comparison to cellulitis in Taiwan: a nationwide population-based case-control study. J Formos Med Assoc.

[9] van Sambeek CHL, van Stigt SF, Brouwers L, Bemelman M (2017). Necrotising fasciitis: a ticking time bomb?. BMJ Case Rep.

[10] Plainvert C, Longo M, Seringe E, Saintpierre B, Sauvage E, Ma L (2018). A clone of the emergent Streptococcus pyogenes emm89 clade responsible for a large outbreak in a post-surgery oncology unit in France. Med Microbiol Immunol.

[11] Wong SSY, Yuen KY (2018). The comeback of scarlet fever. EBio Med.

[12] Shannon BA, McCormick JK, Schlievert PM (2019). Toxins and Superantigens of Group A Streptococci. Microbiol Spectr.

[13] Bisno AL, Brito MO, Collins CM (2003). Molecular basis of group A Streptococcal virulence. Lancet Infect Dis.

[14] Courtney HS, Hasty DL, Dale JB (2006). Anti-phagocytic mechanisms of Streptococcus pyogenes: binding of fibrinogen to M-related protein. Mol Microbiol.

[15] De Oliveira DM, Hartley-Tassell L, Everest-Dass A, Day CJ, Dabbs RA, Ve T (2017). Blood group antigen recognition via the Group A Streptococcal m protein mediates host colonization. MBio.

[16] DebRoy S, Li X, Kalia A, Galloway-Pena J, Shah BJ, Fowler VG (2018). Identification of a chimeric emm gene and novel emm pattern in currently circulating strains of emm4 Group A Streptococcus. Microbial Genom.

[17] Olafsdottir LB, Erlendsdottir H, Melo-Cristino J, Weinberger DM, Ramirez M, Kristinsson KG (2014). Invasive infections due to Streptococcus pyogenes: seasonal variation of severity and clinical characteristics, Iceland, 1975 to 2012. Euro Surveill..

[18] Luca-Harari B, Darenberg J, Neal S, Siljander T, Strakova L, Tanna A (2009). Clinical and microbiological characteristics of severe Streptococcus pyogenes disease in Europe. J Clin Microbiol.

[19] Dauby N, Miendje Deyi VY, Delforge V, Martiny D, Mekkaoui L, Hallin M (2019). Streptococcus pyogenes infections with limited emm-type diversity in the homeless population of Brussels, 2016-2018. Int J Infect Dis.

[20] Meehan M, Murchan S, Gavin PJ, Drew RJ, Cunney R (2018). Epidemiology of an upsurge of invasive group A Streptococcal infections in Ireland, 2012-2015. J Infect.

[21] Tuffs SW, Haeryfar SMM, McCormick JK (2018). Manipulation of innate and adaptive immunity by Staphylococcal superantigens. Pathogens.

[22] Berman HF, Tartof SY, Reis JN, Reis MG, Riley LW (2014). Distribution of superantigens in group A streptococcal isolates from Salvador, Brazil. BMC Infect Dis.

[23] Commons R, Rogers S, Gooding T, Danchin M, Carapetis J, Robins-Browne R (2008). Superantigen genes in group A streptococcal isolates and their relationship with emm types. J Med Microbiol.

[24] Proft T, Fraser JD (2003). Bacterial superantigens. Clin Exp Immunol.

[25] Reglinski M, Sriskandan S, Turner CE (2019). Identification of two new core chromosome-encoded superantigens in Streptococcus pyogenes; speQ and speR. J Infect.

[26] Lin JN, Chang LL, Lai CH, Lin HH, Chen YH (2013). Emergence of Streptococcus pyogenes emm102 causing toxic shock syndrome in Southern Taiwan during 2005-2012. PLoS One.

[27] Lu B, Fang Y, Fan Y, Chen X, Wang J, Zeng J (2017). High prevalence of macrolide-resistance and molecular characterization of Streptococcus pyogenes isolates circulating in China from 2009 to 2016. Front Microbiol.

[28] Zeppa JJ, Kasper KJ, Mohorovic I, Mazzuca DM, Haeryfar SMM (2017). Nasopharyngeal infection by Streptococcus pyogenes requires superantigen-responsive vbeta-specific T cells. Proc Natl Acad Sci U S A.

[29] Chalker V, Jironkin A, Coelho J, Al-Shahib A, Platt S, Kapatai G (2017). Genome analysis following a national increase in scarlet fever in England 2014. BMC genomics.

[30] Commons R, Rogers S, Gooding T, Danchin M, Carapetis J, Robins-Browne R (2008). Superantigen genes in group A streptococcal isolates and their relationship with emm types. J med microbiol.

[31] Lintges M, van der Linden M, Hilgers RD, Arlt S, Al-Lahham A, Reinert RR (2010). Superantigen genes are more important than the emm type for the invasiveness of group A Streptococcus infection. J Infect Dis.

[32] Paveenkittiporn W, Nozawa T, Dejsirilert S, Nakagawa I, Hamada S (2016). Prevalent emm types and superantigen gene patterns of group A Streptococcus in Thailand. Epidemiol Infect.

[33] Balaji K, Thenmozhi R, Prajna L, Dhananjeyan G, Pandian SK (2013). Comparative analysis of emm types, superantigen gene profiles and antibiotic resistance genes among Streptococcus pyogenes isolates from ocular infections, pharyngitis and asymptomatic children in South India. Infect Genet Evol.

[34] Liu X, Shen X, Chang H, Huang G, Fu Z, Zheng Y (2009). High macrolide resistance in Streptococcus pyogenes strains isolated from children with pharyngitis in China. Pediatr Pulmonol.

[35] Nelson MM, Waldron CL, Bracht JR (2019). Rapid molecular detection of macrolide resistance. BMC Infect Dis.

[36] Sayyahfar S, Fahimzad A (2015). Antibiotic susceptibility evaluation of group A streptococcus isolated from children with pharyngitis: a study from Iran. Infect Chemother.

[37] Jose JJM, Brahmadathan KN, Abraham VJ, Huang CY, Morens D, Hoe NP (2018). Streptococcal group A, C and G pharyngitis in school children: a prospective cohort study in Southern India. Epidemiol Infect.

[38] Zafar A, Hasan R, Nizamuddin S, Mahmood N, Mukhtar S, Ali F (2016). Antibiotic susceptibility in Streptococcus pneumoniae, Haemophilus influenzae and Streptococcus pyogenes in Pakistan: a review of results from the survey of antibiotic resistance (SOAR) 2002-15. J Antimicrob Chemother.

[39] Masud S, Mirza S, Abbasi S, Usman M, Khan M, Waqar A (2005). Incidence of erythromycin resistance in clinical isolates of Streptococcus pyogenes at AFIP, Rawalpindi. Pak J Pathol.

[40] Memon BA (2007). Erythromycin resistance in Streptococcus Pyogenes group a throat isolates in Sukkurcity. Rawal Med J.

[41] Rizwan M, Bakht J, Bacha N, Ahmad B (2016). In vitro activity of antimicrobial agents against streptococcus pyogenes isolated from different regions of Khyber Pakhtun Khwa Pakistan. Pak J Pharm Sci.

[42] Richter SS, Heilmann KP, Dohrn CL, Beekmann SE, Riahi F, Garcia-de-Lomas J (2008). Increasing telithromycin resistance among Streptococcus pyogenes in Europe. J Antimicrob Chemother.

[43] Alfaresi MS (2010). Group A streptococcal genotypes from throat and skin isolates in the United Arab Emirates. BMC Res Notes.

[44] Mihaila-Amrouche L, Bouvet A, Loubinoux J (2004). Clonal spread of emm type 28 isolates of Streptococcus pyogenes that are multiresistant to antibiotics. J Clin Microbiol.

[45] Abraham T, Sistla S (2016). Identification of Streptococcus pyogenes - phenotypic tests vs molecular assay (spy1258PCR): a comparative study. J Clin Diag Res.

[46] Luca-Harari B, Ekelund K, van der Linden M, Staum-Kaltoft M, Hammerum AM, Jasir A (2008). Clinical and epidemiological aspects of invasive Streptococcus pyogenes infections in Denmark during 2003 and 2004. J Clin Microbiol.

[47] Engel ME, Muhamed B, Whitelaw AC, Musvosvi M, Mayosi BM, Dale JB (2014). Group A streptococcal emm type prevalence among symptomatic children in Cape Town and potential vaccine coverage. Pediat Infect Dis J.

[48] Chochua S, Metcalf BJ, Li Z, Rivers J, Mathis S, Jackson D (2017). Population and Whole Genome Sequence Based Characterization of Invasive Group A Streptococci Recovered in the United States during 2015. MBio.

[49] Rudolph K, Bruce MG, Bruden D, Zulz T, Reasonover A, Hurlburt D (2016). Epidemiology of invasive group A streptococcal disease in Alaska, 2001 to 2013. J Clin Microbiol.

[50] Eisner A, Leitner E, Feierl G, Kessler HH, Marth E (2006). Prevalence of emm types and antibiotic resistance of group A streptococci in Austria. Diag Microbiol Infect Dis.

[51] Mathur P, Bhardwaj N, Mathur K, Behera B, Gupta G, Kapil A (2014). Clinical and molecular epidemiology of beta-hemolytic streptococcal infections in India. J Infect Dev Ctries.

[52] Bergmann R, Nerlich A, Chhatwal GS, Nitsche-Schmitz DP (2014). Distribution of small native plasmids in Streptococcus pyogenes in India. Int J Med Microbiol.

[53] Sagar V, Bergmann R, Nerlich A, McMillan DJ, Nitsche-Schmitz DP, Fulde M (2014). Differences in virulence repertoire and cell invasive potential of group A Streptococcus emm1-2 in comparison to emm1 genotype. Int J Med Microbiol.

[54] Lamb LE, Siggins MK, Scudamore C, Macdonald W, Turner CE, Lynskey NN (2018). Impact of contusion injury on intramuscular emm1 group A Streptococcus infection and lymphatic spread. Virulence.

[55] Ikebe T, Tominaga K, Shima T, Okuno R, Kubota H, Ogata K (2015). Increased prevalence of group A Streptococcus isolates in Streptococcal toxic shock syndrome cases in Japan from 2010 to 2012. Epidemiol Infect.

[56] Michaelsen TE, Andreasson IK, Langerud BK, Caugant DA (2011). Similar superantigen gene profiles and superantigen activity in norwegian isolates of invasive and noninvasive group A Streptococci. Scand J Immunol.

[57] Vahakuopus S, Vuento R, Siljander T, Syrjanen J, Vuopio J (2012). Distribution of emm types in invasive and noninvasive group A and G streptococci. Eur J Clin microbiol Infect Dis.

[58] Reglinski M, Sriskandan S (2014). The contribution of group A streptococcal virulence determinants to the pathogenesis of sepsis. Virulence.

[59] Mathur P, Bhardwaj N, Mathur K, Behera B, Gupta G, Kapil A (2014). Clinical and molecular epidemiology of beta-hemolytic streptococcal infections in India. J Infect Dev Ctries.

[60] Imohl M, Fitzner C, Perniciaro S, van der Linden M (2017). Epidemiology and distribution of 10 superantigens among invasive Streptococcus pyogenes disease in Germany from 2009 to 2014. PLoS One.

[61] Davies MR, Holden MT, Coupland P, Chen JH (2015). Emergence of scarlet fever Streptococcus pyogenes emm12 clones in Hong Kong is associated with toxin acquisition and multidrug resistance. Nat Genet.

[62] You Y, Davies MR, Protani M, McIntyre L, Walker MJ, Zhang J (2018). Scarlet fever epidemic in china caused by Streptococcus pyogenes serotype M12: epidemiologic and molecular analysis. EBio Med.

[63] Walker MJ, Brouwer S, Forde BM, Worthing KA, McIntyre L, Sundac L (2019). Detection of epidemic scarlet fever group A Streptococcus in Australia. Clin Infect Dis.

